# A deep learning approach for ^18^F-FDG PET attenuation correction

**DOI:** 10.1186/s40658-018-0225-8

**Published:** 2018-11-12

**Authors:** Fang Liu, Hyungseok Jang, Richard Kijowski, Gengyan Zhao, Tyler Bradshaw, Alan B. McMillan

**Affiliations:** 1 Departments of Radiology, University of Wisconsin School of Medicine and Public Health, 600 Highland Avenue, Madison, WI 53705-2275 USA; 2 Medical Physics, University of Wisconsin School of Medicine and Public Health, 600 Highland Avenue, Madison, WI 53705-2275 USA; 3 Departments of Radiology, Wisconsin Institutes for Medical Research, 1111 Highland Avenue, Madison, WI 53705-2275 USA

**Keywords:** Deep learning, PET, CT, MRI, PET/MR, PET/CT, Attenuation correction

## Abstract

**Background:**

To develop and evaluate the feasibility of a data-driven deep learning approach (deepAC) for positron-emission tomography (PET) image attenuation correction without anatomical imaging. A PET attenuation correction pipeline was developed utilizing deep learning to generate continuously valued pseudo-computed tomography (CT) images from uncorrected ^18^F-fluorodeoxyglucose (^18^F-FDG) PET images. A deep convolutional encoder-decoder network was trained to identify tissue contrast in volumetric uncorrected PET images co-registered to CT data. A set of 100 retrospective 3D FDG PET head images was used to train the model. The model was evaluated in another 28 patients by comparing the generated pseudo-CT to the acquired CT using Dice coefficient and mean absolute error (MAE) and finally by comparing reconstructed PET images using the pseudo-CT and acquired CT for attenuation correction. Paired-sample *t* tests were used for statistical analysis to compare PET reconstruction error using deepAC with CT-based attenuation correction.

**Results:**

deepAC produced pseudo-CTs with Dice coefficients of 0.80 ± 0.02 for air, 0.94 ± 0.01 for soft tissue, and 0.75 ± 0.03 for bone and MAE of 111 ± 16 HU relative to the PET/CT dataset. deepAC provides quantitatively accurate ^18^F-FDG PET results with average errors of less than 1% in most brain regions.

**Conclusions:**

We have developed an automated approach (deepAC) that allows generation of a continuously valued pseudo-CT from a single ^18^F-FDG non-attenuation-corrected (NAC) PET image and evaluated it in PET/CT brain imaging.

## Background

Positron-emission tomography (PET) is a non-invasive imaging modality that provides direct imaging of biomarkers for physiology through the use of radiolabeled molecules, such as ^18^F-fluorodeoxyglucose (FDG) to assess glucose metabolism. PET activity is observed by detecting pairs of coincident gamma rays emitted from the PET tracer, sorted into sinograms, and reconstructed into a volumetric image. Knowledge of the tissue-dependent attenuation (typically Compton scatter) that gamma rays undergo is crucial to achieve quantitatively accurate PET reconstruction. In a typical PET scanner, the attenuation map (or μ-map) is obtained by performing additional anatomic imaging, which typically increases patient ionizing radiation dose. In a combined PET/CT system, an acquired CT image is used to generate an attenuation map for 511 keV photons by simple piecewise scaling of a CT image [1]. However, the attenuation map is estimated using a single snapshot in time which does not reflect misregistration due to patient motion between the acquisition of the CT and PET scans. In simultaneous PET/MR systems, estimation of the required attenuation map is based on MR images (which does not increase patient ionizing radiation dose) and is particularly challenging because the bone, the tissue with the largest attenuation coefficient, is not visible with positive contrast under typical MR acquisitions. Consequently, the bone is often ignored or estimated using atlas registration methods [2]. Specialized MRI acquisitions using a short echo time (STE), ultrashort echo time (UTE), or zero echo time (ZTE) can be implemented to allow the measurement of the rapidly decaying MR signal in the bone tissue to estimate the bone [3–6]. Unfortunately, UTE and ZTE acquisitions provide little clinical value compared to the conventional diagnostic imaging sequences. Additionally, even with advanced acquisitions, bony structure and air often remain difficult to distinguish, and errors remain in attenuation calculation [3, 7, 8].

Machine learning approaches have previously been proposed to estimate attenuation maps. A few pilot studies utilizing neural network methods have shown promising results in PET/MR imaging [9]. These studies typically rely on the inputs of T1-weighted MR images [10, 11], ultrashort-echo time MR images and transmission images [12] to estimate pseudo-CTs which can be applied to PET attenuation correction. More recently, deep learning approaches using Convolutional Neural Networks (CNN) have been applied to medical imaging with successful implementations across a diverse range of applications [13]. Deep learning methods are more advanced forms of neural networks and utilizing many levels of network structures capable of learning image features by a series of image convolution processes [14]. One recent study used deep learning to generate discrete pseudo-CTs for PET/MR attenuation correction using a single T1-weighted head image, which significantly reduced PET error in an evaluation dataset of ten brain images in comparison with vendor-provided segmentation- and atlas-based methods [15]. However, in all previous studies, besides PET data, additional acquisitions of either MR images or transmission images were required inputs. In this study, we propose a novel automated PET image attenuation correction approach, deepAC, that allows generation of a continuously valued pseudo-CT using only a non-attenuation-corrected (NAC) PET image as input. To the best of our knowledge, this proposed method is the first pilot study performing PET attenuation correction utilizing a self-regularized approach. The feasibility of this new approach is demonstrated in the human brain utilizing ^18^F-FDG PET/CT datasets and is compared to reconstruction utilizing the acquired CT. Given the favorable results of deepAC, we expect deep learning-based approaches to have a substantial impact to maintain accurate PET reconstruction while reducing the ionizing radiation dose and increasing the resilience to subject motion.

## Methods

### Convolutional encoder-decoder architecture

**Fig. 1:**
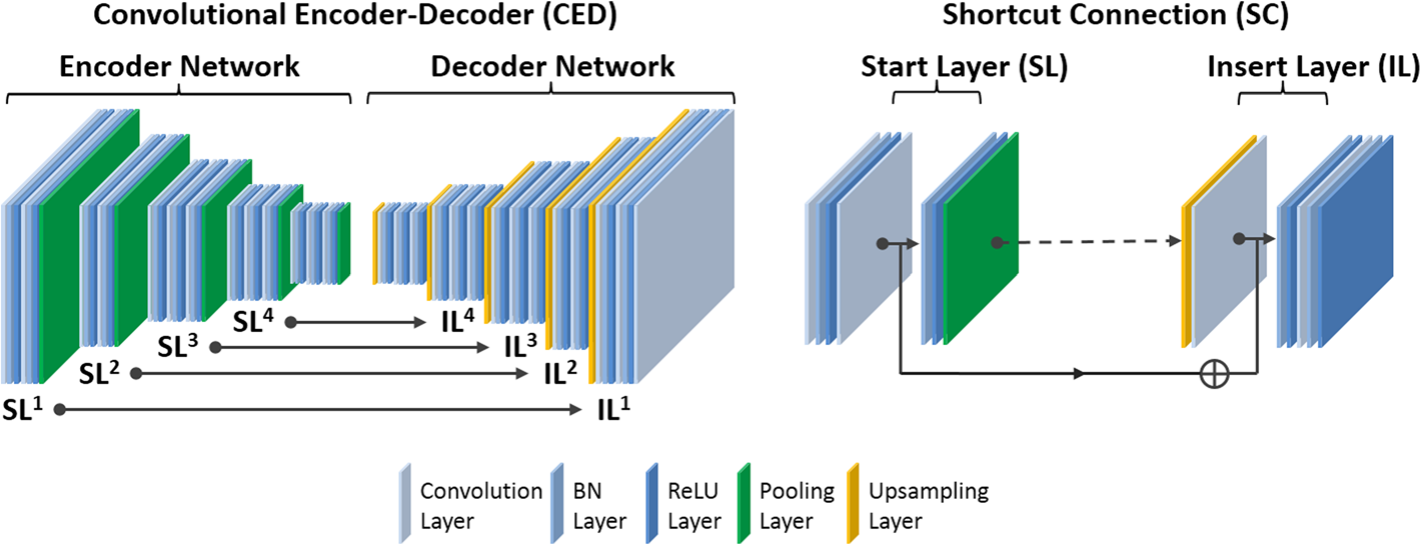
Schematic illustration of convolutional encoder-decoder in this study. This network consists of multiple symmetrical shortcut connection (SC) from the start layer (SL) in the encoder to the insert layer (IL) in the decoder. The insertion of SC follows the strategy of the deep residual network described in Reference [22]

The key component of our proposed method is a deep convolutional encoder-decoder (CED) network, which is capable of mapping the NAC PET image into a pixel-wise continuously valued pseudo-CT image. The CED framework was modified based on the network structure used in a previous study for generating discrete three-class pseudo-CT in PET/MR attenuation correction [15]. A schematic demonstration of this deep learning network is shown in Fig. 1. The network features a connected encoder network and a decoder network. The encoder is designed to compress input image data while detecting robust and spatially invariant features. The Visual Geometry Group 16 (VGG16) network [16] was used as an encoder because this network has been proven to be efficient in capturing image features in object recognition and to be effective in CED-based medical image applications [7, 15, 17, 18]. Each unit layer in the VGG16 encoder consists of 2D convolution layer with a set of 2D filters, batch normalization (BN) [19], rectified linear unit (ReLU) activation [20], and followed by a max-pooling for the reduction of data dimensions. An increased number of 2D filters from 64 to 512 were generated by convolution layers from the first unit layer to the last in the encoder network to efficiently extract the input image features. Batch normalization was used to reduce the internal image covariate shift during network training so as to increase the training efficiency [19]. The ReLU activation function was used to perform non-linear transformation of the input image features so as to increase the network capability for learning a complex information. Max-pooling used a 2 × 2 window and stride 2 which leads to sub-sampled feature maps with a size reduction factor of 2. This unit layer was repeated 13 times in the VGG16 configuration to achieve sufficient data compression.

To reconstruct pixel-wise continuously valued CT images, a decoder network is applied directly after the encoder network. This decoder network is the reverse process of the encoder and consists of mirrored layers from the VGG16 network. The max-pooling layer in the decoder is replaced by an un-pooling layer (i.e., upsampling) where the image features were upsampled using bilinear interpolation. At the end of the decoder network, an additional 2D convolutional layer with one 2D filter was added to synthesize the output pseudo-CT images based on the decoded image features from the upstream decoder network.

In contrast to the network used in pseudo-CT generation in Reference [15], the proposed network also features shortcut connections (SC) which are used to forwardly pass the image features from an encoder network to a decoder network. As shown in the current study, synthesis of the continuously valued CT images requires preservation of additional rich image features compared to the discretely valued CT images in Reference [15], for which the added shortcut connections are valuable [21, 22]. Since it is well known that training a deep network is challenging due to the vanishing gradient problem [21], shortcut connections are helpful for enabling efficient training of networks with a large number of layers and complex structure. As shown in Fig. 1, the shortcut connections occur symmetrically in multiple layers in the networks and link feature maps by adding ones from the encoder network to the ones in the decoder network element-wise. A total of four shortcut connections were created between the network layers, and one additional shortcut connection was also generated from the input image directly to the output image. For each shortcut connection, the layer insertion (Fig. 1) to transfer feature maps occurred prior to the BN and ReLU activation in the unit layer of the decoder. This strategy was also described as the full pre-activation version of deep residual network design [22].

### Proposed PET processing pipeline

**Fig. 2:**
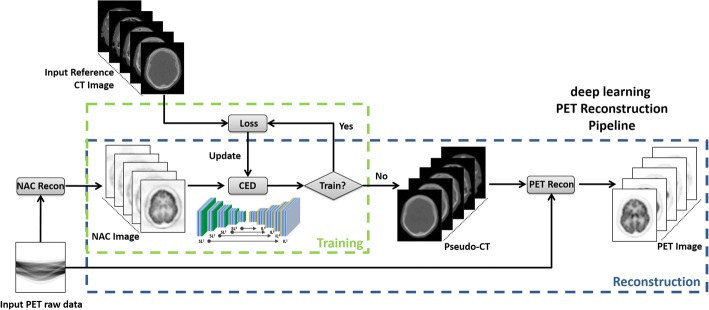
Schematic illustration of deepAC. The process consists of a training phase and a reconstruction phase. The training phase is first performed with NAC and co-registered CT data, after which the well-trained network is fixed and ready for generating pseudo-CTs for new PET data in the reconstruction phase

The proposed processing pipeline consists of two independent phases for training retrospective data and reconstructing new data, respectively. In the training phase (Fig. 2), the training data for the proposed CED network consists of NAC PET images as input and reference non-contrast-enhanced CT data. For each training dataset, NAC PET and co-registered CT images were first offset to positive values and then scaled by pixel intensity of 6000 Bq/ml and 2000 Hounsfield unit (HU), respectively, to ensure a similar dynamic range. 3D NAC PET and CT images were cropped to enclose the image object with a minimally sized bounding box to remove redundant background prior to deep learning training. 2D axial slices from the 3D volumetric NAC and CT images were used as inputs to the deep learning network. All 2D input images were first transformed pixel-wise using a Softsign activation function in order to maintain a compact dynamic range and resampled to a matrix size of 200 × 180 using bilinear interpolation before being used as input to the CED. The encoder and decoder network weights were initialized using a normal distribution centered on 0 with a standard deviation varying according to the number of input weights. This initialization scheme was described in Reference [23]. Network updating used a gradient-based optimization algorithm (ADAM) [24] with a fixed and empirically selected learning rate of 0.001. The CED network iteratively estimated continuous CT images and compared them to the reference real CT data. The data consistency between estimated and real CT image was ensured by using mean squared error (MSE) as an image loss objective function where the loss was calculated in a mini-batch of 12 images in each iteration given our current hardware setup. The network training was performed for 20,000 iterations which corresponded to 50 epochs for our training data to achieve training loss convergence.

Once the training phase was complete, the CED network at the epoch with the least image loss was fixed and was used for generating continuous pseudo-CT for new PET data, which were subsequently used for PET reconstruction (Fig. 2).

In this study, the proposed framework was implemented in a hybrid computing environment involving Python (version 2.7, Python Software Foundation, Wilmington, DE, USA) and MATLAB (version 2013a, MathWorks, Natick, MA, USA). The image processing and analysis steps were performed in MATLAB. The CED network was coded using the Keras package with Tensorflow deep learning libraries as the computing backend [25].

### Image datasets for training and evaluation

Our study was performed in compliance with the Health Insurance Portability and Accountability Act (HIPAA) regulations and with approval from our Institutional Review Board (IRB). Data collection for training and evaluation of the proposed deepAC method was performed utilizing an IRB-approved protocol for retrospective analysis. Subject eligibility criteria included any subjects who underwent a whole-body ^18^F-FDG PET and a non-contrast CT scan on the Discovery PET/CT 710 scanner (GE Healthcare, Waukesha, WI, USA) at our institution in 2016. Images from the first 140 consecutive patients meeting our eligibility criteria were retrieved from PACS in chronological order. Subjects were excluded from the training and evaluation if visual inspection revealed a spatial mismatch between NAC PET and CT images due to subject motion, which resulted in the removal of 12 subjects from the dataset. The dataset included both arms-up and arms-down positioning, with no discrimination upon either type of positioning. Subjects used for training and testing had a median age of 65 (range, 19–92) with 52 males and 76 females. Per our institution’s FDG PET protocol, subjects fasted for at least 6 h before the exam, had blood glucose less than 200 mg/dl, and were injected with 0.14 mCi/kg of tracer. Scanning began 56.5 ± 2.5 min after injection of FDG, with 3 min PET acquisitions per bed position. CT images were obtained with the following acquisition/reconstruction settings: 1.37 mm transaxial voxel dimensions, 5 mm slice thicknesses with 3.27 mm interslice spacing, 140 kVp, automatic exposure control with GE noise index of 25, and 0.52 helical pitch. NAC PET images were reconstructed offline (PET Toolbox, GE Healthcare) without attenuation correction and without time-of-flight, using the following parameters: 256 × 256 matrix, 700 × 700 mm^2^ field of view, OSEM reconstruction algorithm, 24 iterations, 3 subsets, SharpIR, and 4-mm post filter.

For training and evaluation, image data from the head was selectively used from each whole-body PET/CT dataset. The CED network was trained using 100 randomly selected subjects and evaluated in the remaining 28 subjects. All training and testing were performed on a desktop computer running a 64-bit Linux operating system with an Intel Xeon W3520 quad-core CPU, 12 GB DDR3 RAM, and a Nvidia Quadro K4200 graphic card (1344 CUDA cores, 4 GB GDDR5 RAM).

### Pseudo CT generation and evaluation

After a NAC PET image is fed into the network, the output of the CED is scaled back to HUs. Analysis of pseudo-CT accuracy was performed on 28 subjects not included in the training phase of the CED network. Pixel-wise Dice coefficient, a similarity measure ranging from 0 to 1 that describes the overlap between two labels, was calculated for air, soft tissue, and bone. For calculation of Dice coefficients, the continuous pseudo-CT and real CT images were discretized by thresholding as follows: bone if HU > 300, air if HU < − 400, otherwise soft tissue. Additionally, the mean absolute error (MAE) between the generated pseudo-CT and real CT images was evaluated in each subject. Statistical analysis was performed using MATLAB.

### PET image reconstruction

Offline PET reconstruction (PET Toolbox, GE Healthcare) was performed with a pseudo-CT image generated by deepAC and the acquired CT image for attenuation correction. PET reconstruction parameters were 256 × 256 matrix, 700 × 700 mm^2^ field of view, TOF-OSEM reconstruction algorithm, 24 iterations, 3 subsets, SharpIR, model-based scatter correction, and 4-mm post filter.

### Evaluation of PET quantification

Evaluation of reconstructed PET image quality in deepAC was performed with the 28 testing subjects. PET images reconstructed using the deepAC-based attenuation correction were compared to those reconstructed using the acquired CT-based attenuation correction (CTAC). Pixel-wise error maps were obtained using the percentage error:

1 $$ \mathrm{Err}=\frac{I_{\mathrm{deepAC}}-{I}_{\mathrm{CTAC}}}{I_{\mathrm{CTAC}}}\times 100\% $$

where *I*_deepAC_ and *I*_CTAC_ are the PET image intensity (Bq/ml) from deepAC and CTAC, respectively. We also calculated the absolute value of Err as the absolute percentage error. Region of interest (ROI) analysis was performed using the IBASPM parcellation software with a PET brain atlas to compute ROI-level errors in 21 brain regions [26]. The data normality assumption was checked, and paired-sample *t* tests were used to perform the pairwise comparison for the PET activity in these brain regions using deepAC and CTAC. Statistical analysis was performed using MATLAB and R (R Development Core Team) with statistical significance defined as a *p* < 0.05 with Bonferroni correction for minimizing type-I error (equivalent to uncorrected *p* < 0.0024).

## Results

**Fig. 3:**
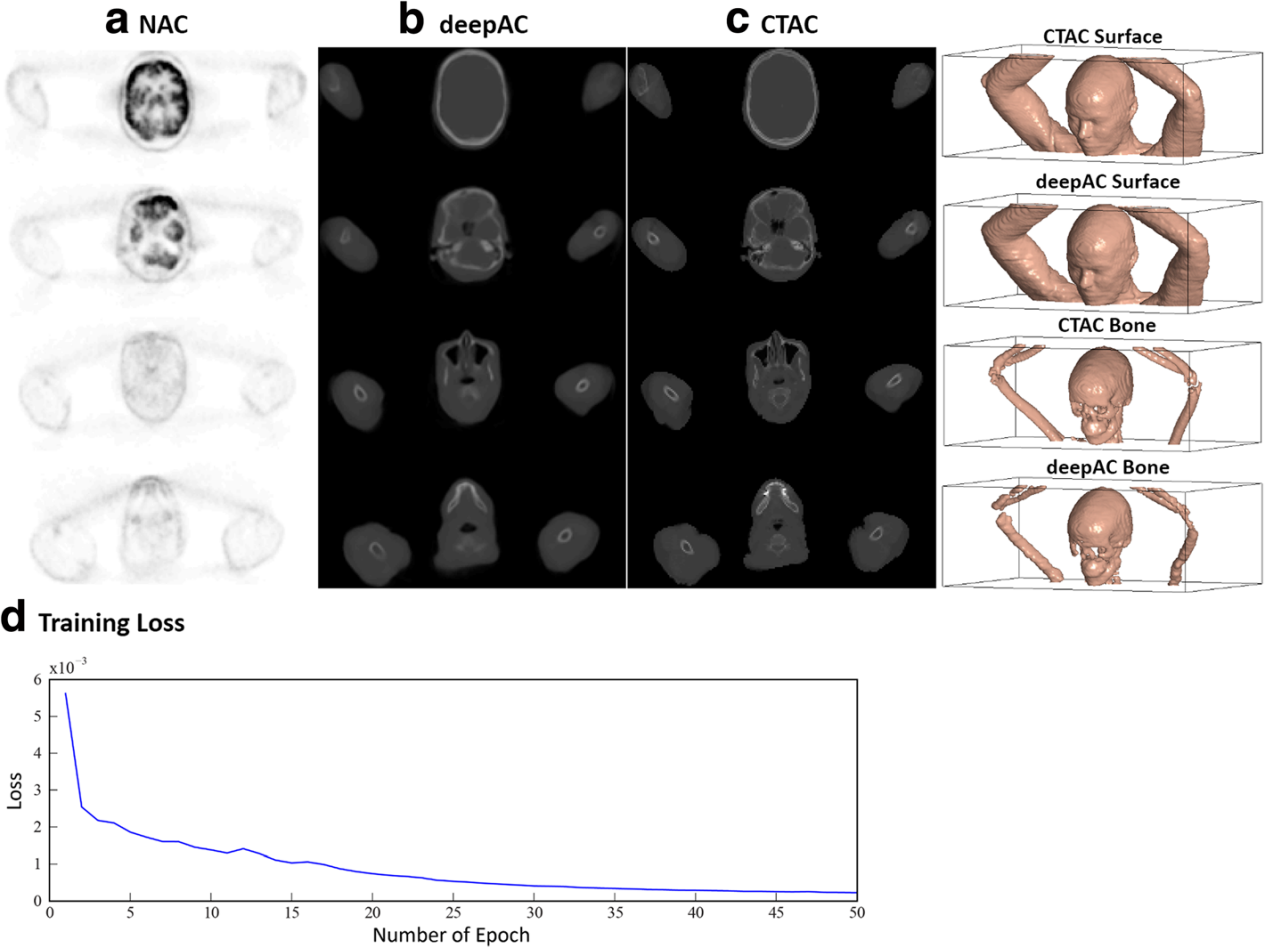
Example of pseudo-CT image from deepAC. Multiple axial slices from **a** the input NAC PET image, **b** the pseudo-CT generated using deepAC, and **c** the acquired CT. The 3D surface and bone model indicate a high similarity between the acquired CT and pseudo-CT. The surface and bone were rendered using a HU value of − 400 and 300, respectively. The training loss curve is shown in **d**

An example of an acquired NAC PET image, deepAC pseudo-CT and real CT for a 48-year-old female subject is shown in Fig. 3. As shown in this figure, deepAC was able to identify the air, skull, and soft tissue in the NAC PET images and synthesize continuous CT values for distinct tissue types. The total training stage required approximately 30 h, whereas generating a single pseudo-CT output using the trained model required approximately 0.5 min. The training loss curve is shown in Fig. 3. The curve indicated a successful training convergence where the training loss gradually decreased prior to 40 epochs and reached a plateau after 40 epochs. Dice coefficients for the evaluation subset (*n* = 28) comparing the pseudo-CT to the real CT were high for air, 0.80 ± 0.02; soft tissue, 0.94 ± 0.01; and bone, 0.75 ± 0.03. The MAE between pseudo-CT and real CT was 111 ± 16 HU for all subjects with a range between 88 and 157 HU.

**Fig. 4:**
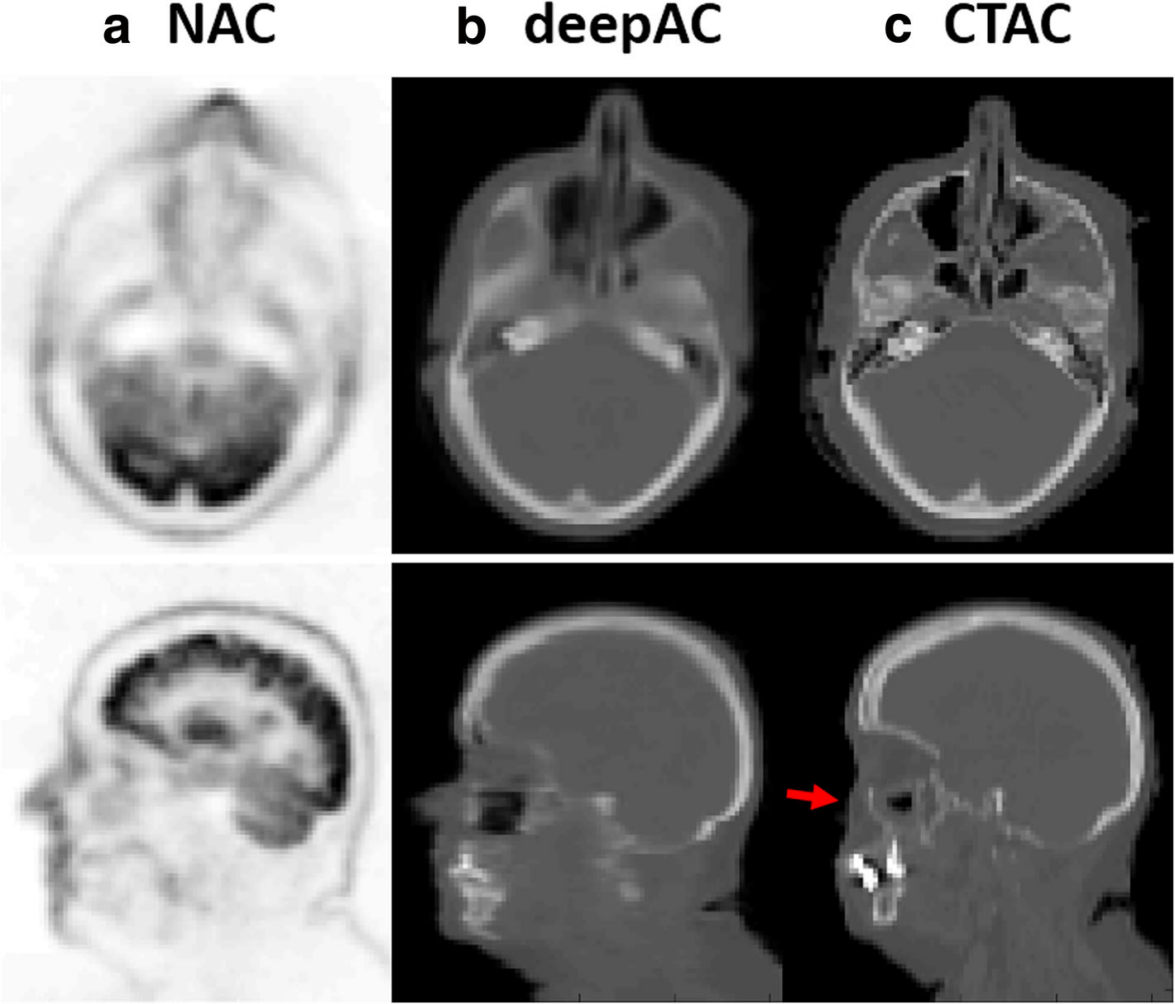
Example of pseudo-CT image from a non-compliant subject. Axial and sagittal slices from **a** the input NAC PET image, **b** the pseudo-CT generated using deepAC, and **c** the acquired CT. Note that there is a noticeable movement between PET and CT scans (red arrow). The generated pseudo-CT from deepAC is free from subject motion since it is directly obtained from PET data

Another example of an acquired NAC PET image, deepAC pseudo-CT and real CT are shown in Fig. 4 for a non-compliant patient who exhibited significant movement between the PET and CT scans. This 52-year-old male subject is one of the cases excluded from training and PET evaluation. The misregistration can be clearly identified between NAC PET and real CT images in the axial and sagittal slices (red arrow) at the same image location. In contrast, the pseudo-CT generated from deepAC is free from misregistration with respect to the NAC PET data.

**Fig. 5:**
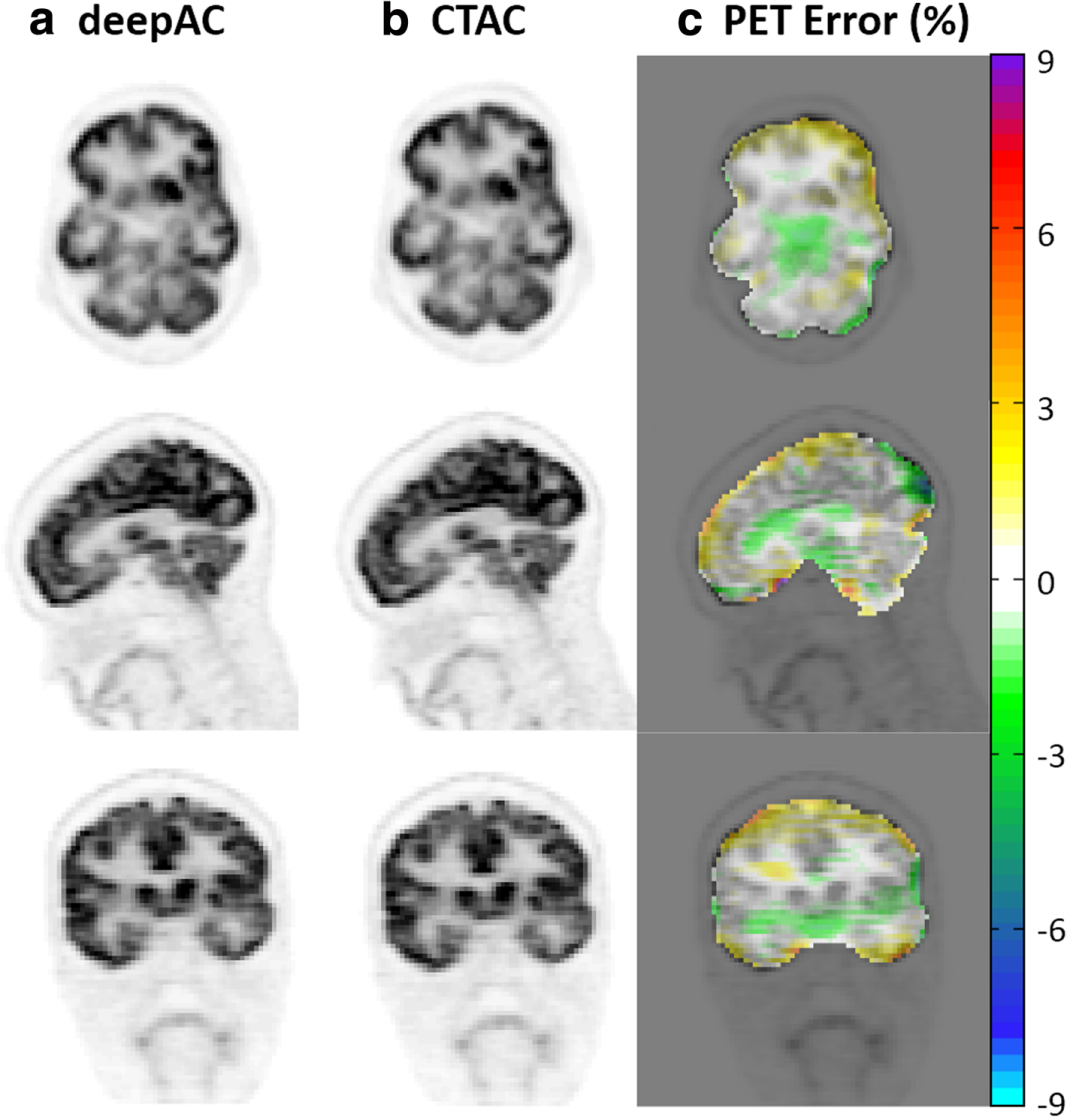
PET reconstruction using **a** deepAC and **b** acquired CT-based attenuation correction (CTAC) for a 48-year-old female subject. **c** Relative error was calculated using the PET image reconstructed using CTAC. Low reconstructed PET error is observed by using the proposed deepAC approach

Figure 5 shows the reconstructed PET image for the subject of Fig. 3, utilizing the pseudo- and real CT for attenuation correction, respectively, as well as pixel-wise relative difference images between these. As seen in Fig. 5, deepAC results in PET error of less than 1% in most of the brain regions.

**Fig. 6:**
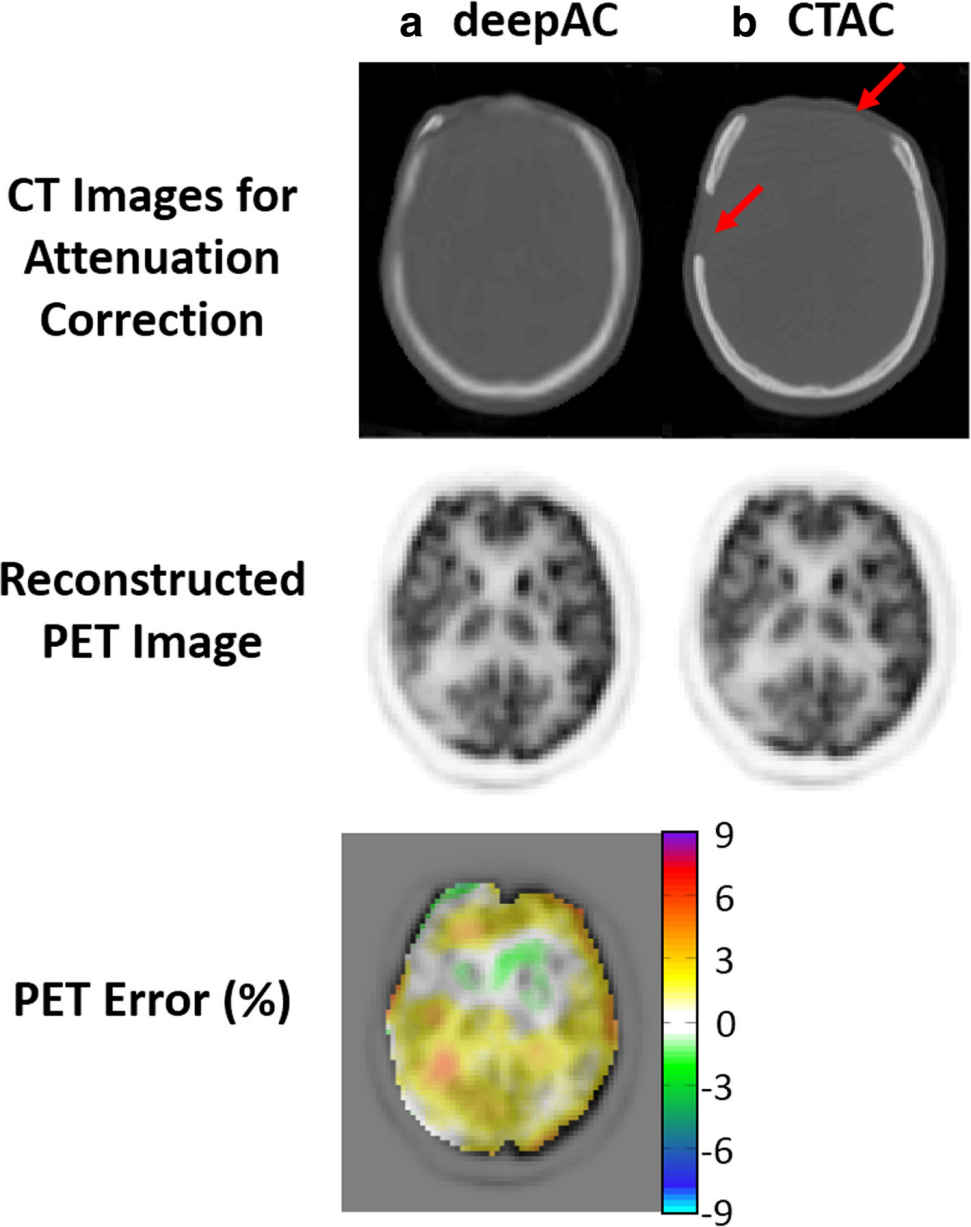
PET reconstruction using **a** deepAC and **b** acquired CT-based attenuation correction (CTAC) for an 80-year-old female with a significant right and frontal skull abnormality. The missing parts of the skull were indicated by red arrows in real CT image. Low reconstructed PET error is observed by using the proposed deepAC approach given the case of skull abnormality

A challenging case for deepAC is shown in Fig. 6 for an 80-year-old female with a significant right and frontal skull abnormality. The generated pseudo-CT was able to predict the parts of missing skull in the forehead, indicated by the red arrows in the real CT image. The average reconstructed PET error for this subject in all brain pixels is 0.87%. Despite significant skull abnormalities relative to typical patients, PET reconstruction error was maintained at a low level utilizing deepAC.

**Fig. 7:**
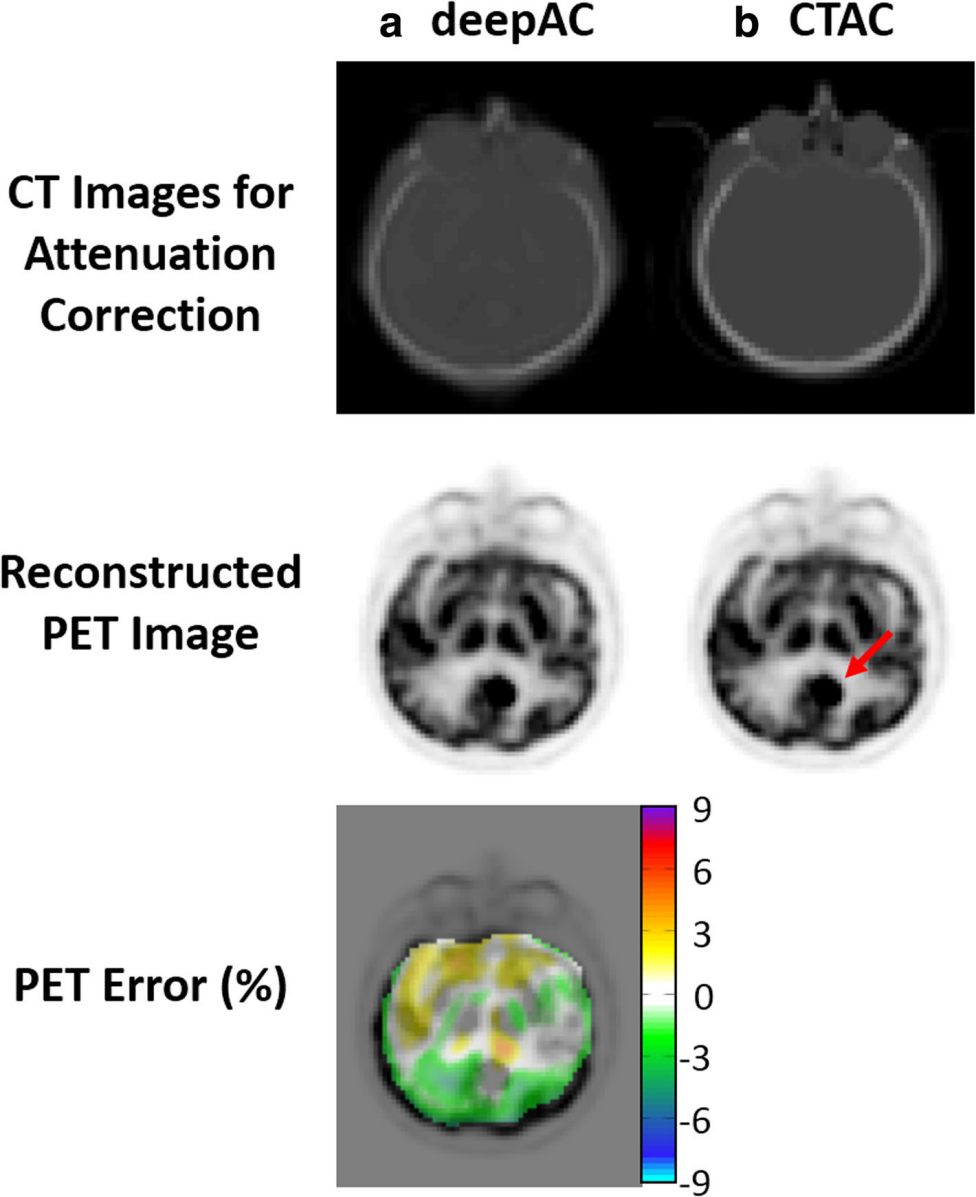
PET reconstruction using **a** deepAC and **b** acquired CT-based attenuation correction (CTAC) for a 59-year-old male with a brain tumor. The tumor region was indicated by a red arrow in CTAC PET image. Low reconstructed PET error is observed by using the proposed deepAC approach given the presence of brain metastasis

Another case for deepAC is shown in Fig. 7 for a 59-year-old male with a brain tumor, indicated by the red arrow in the reconstructed PET image using CTAC. The average reconstructed PET error for this subject in all brain pixels is 0.52%. Despite the presence of brain tumor which was rare in our dataset, deepAC PET reconstruction was able to correctly identify tumor region and the global error was maintained at a low level.

Table 1 provides an average error, standard deviation, and *p* values from paired *t* tests within different brain regions across all 28 subjects. Reconstructed PET error on the ROI level showed that deepAC provided average PET errors below 1% for 15 out of 21 regions and below 2% for all regions tested. Paired *t* tests showed that for 14 out of 21 ROIs, mean PET activity was not significantly different (*p* > 0.05) between the deepAC approach and the reference PET (CTAC) image. For the ROIs that were significantly different, the average signed differences in PET values were small, which included left parietal lobe (*p* = 0.01, average difference = − 1.7%), right parietal lobe (*p* = 0.005, average difference = − 1.92%), left occipital lobe (*p* = 0.01, average difference = − 1.78%), right occipital lobe (*p* = 0.004, average difference = − 1.92%), right putamen (*p* = 0.009, difference = − 0.74%), right globus pallidus (*p* = 0.013, difference = − 0.56%), and left cingulate region (*p* = 0.049, difference = − 0.5%). Note that when corrected for multiple comparisons using a Bonferroni corrected significance level (*p* < 0.0024), none of the region-wise differences remained significant.

**Table 1:** Image error (mean ± standard deviation (minimum, maximum)) relative to CT attenuation correction of PET images reconstructed utilizing deepAC in various brain regions of 28 subjects and *p* values from paired *t* tests. *p* < 0.0024 is defined as the Bonferroni corrected significance level

Brain regions	deepAC error (%)	deepAC absolute error (%)	*p* value (deepAC vs CTAC)
Frontal lobe left	− 1.04 ± 2.35 (− 6.67, 3.32)	2.43 ± 1.57 (0.65, 6.67)	0.18
Frontal lobe right	− 1.15 ± 2.56 (− 6.61, 4.06)	2.59 ± 1.69 (0.82, 6.62)	0.15
Temporal lobe left	− 0.79 ± 1.70 (− 4.45, 2.22)	2.11 ± 0.94 (1.07, 4.67)	0.18
Temporal lobe right	− 0.73 ± 1.99 (− 4.77, 2.68)	2.32 ± 0.96 (1.10, 4.88)	0.055
Parietal lobe left	− 1.70 ± 2.25 (− 5.56, 2.22)	2.52 ± 1.63 (0.51, 5.56)	0.01
Parietal lobe right	− 1.92 ± 2.38 (− 5.60, 2.28)	2.79 ± 1.61 (0.69, 5.60)	0.005
Occipital lobe left	− 1.78 ± 1.95 (− 6.24, 1.40)	2.38 ± 1.43 (0.69, 6.26)	0.01
Occipital lobe right	− 1.92 ± 2.15 (− 6.12, 2.81)	2.75 ± 1.25 (0.89, 6.12)	0.004
Cerebellum left	− 0.22 ± 1.62 (− 3.86, 2.68)	1.70 ± 0.76 (0.67, 3.94)	0.154
Cerebellum right	− 0.27 ± 1.78 (− 3.79, 2.83)	1.78 ± 0.85 (0.54, 3.79)	0.146
Brainstem	0.69 ± 1.79 (− 3.20, 3.81)	1.77 ± 0.88 (0.74, 3.81)	0.354
Caudate nucleus left	0.37 ± 1.71 (− 3.38, 3.69)	1.50 ± 0.85 (0.43, 3.69)	0.613
Caudate nucleus right	0.32 ± 1.64 (− 3.47, 3.21)	1.33 ± 0.86 (0.33, 3.47)	0.451
Putamen left	− 0.67 ± 1.58 (− 3.80, 2.31)	1.41 ± 1.00 (0.24, 3.80)	0.115
Putamen right	− 0.74 ± 1.53 (− 3.94, 2.08)	1.40 ± 1.02 (0.27, 3.94)	0.009
Thalamus left	− 0.07 ± 1.59 (− 3.90, 3.05)	1.40 ± 0.88 (0.31, 3.91)	0.172
Thalamus right	0.00 ± 1.56 (− 4.04, 3.27)	1.34 ± 0.91 (0.20, 4.04)	0.283
Globus pallidus left	− 0.39 ± 1.51 (− 3.71, 3.11)	1.25 ± 0.91 (0.12, 3.71)	0.056
Globus pallidus right	− 0.56 ± 1.41 (− 3.83, 2.35)	1.24 ± 0.92 (0.29, 3.83)	0.013
Cingulate region left	− 0.50 ± 1.68 (− 4.02, 2.70)	1.61 ± 0.95 (0.35 4.02)	0.049
Cingulate region right	− 0.45 ± 1.63 (− 3.81, 2.28)	1.58 ± 0.87 (0.35, 3.81)	0.057
All regions	− 0.64 ± 1.99 (− 4.18, 2.22)	1.74 ± 0.94 (0.29, 4.20)	

## Discussion

In this study, we demonstrated the feasibility of a new approach utilizing only ^18^F-FDG NAC PET images input into a deep learning model trained with PET/CT data to generate pseudo-CT images that can be used to perform quantitatively accurate PET-only imaging. For all ROIs tested, the percentage error in PET reconstruction was less than 2% on average, with the vast majority less than 1% on average. For individual subject data, the maximal percent error in any single region was 6.7%. When compared with CTAC reconstruction, this provided a statistically significant different quantitative PET result in only seven of the regions studied (yet still with an error of less than 2%). After correcting for multiple comparisons (Bonferroni correction), these differences were no longer statistically significant. Given the large training size of 100 subjects, these findings suggest that deep learning approaches can be leveraged to provide quantitatively accurate PET without the acquisition of a CT image. Given the inclusion of anatomically abnormal subjects into the evaluation dataset, e.g. as shown in Figs. 6 and 7, it can be expected that state-of-the-art deep learning approaches, such as the demonstrated herein, can indeed be robust to individual patient variations. With an increased number of training datasets, particularly datasets that encompass the range of variability and abnormalities present, this robustness would be expected to increase. Finally, the extension of deepAC to other regions outside the brain is expected to be valuable. However, we expect to require body-region-specific models such that models can be trained to best match the features of specific anatomical regions.

Reconstruction-based approaches have been previously proposed to synthesize μ-maps directly from PET data [27]. Recent embodiments of these approaches have demonstrated quantitatively accurate PET images with approximately 2–7% error [28, 29]. While these techniques use complicated reconstruction algorithms, they are also likely to benefit from the application of a deep learning approach such as deepAC, to augment or constrain data.

The applications of deepAC are expected to be numerous. In particular, methods that do not require the acquisition of a CT could be used to reduce the ionizing radiation exposure for patients undergoing repeated PET imaging studies. Depending on the protocol used, the patient exposure from CT is typically comparable to the whole-body equivalent PET exposure [30]. For pediatric and pregnant patients, this would significantly reduce ionizing radiation exposure. Other potential applications include management of misregistration between the PET and CT, where if significant motion occurs between the CT and PET acquisitions (e.g., Fig. 4), deepAC would be able to accurately compensate for this movement. The current implementation of deepAC does not necessarily help for fully non-compliant patients with significant motion during PET acquisition. One possible solution for this approach is to use deepAC to generate multi-phase dynamic pseudo-CTs to capture more intensive and dynamic motion. However, this would require additional prospective experimental design to obtain both dynamic PET and dynamic CT data. Additional applications of deepAC include utilization in research-only PET studies where CT adds minimal value beyond attenuation correction. Finally, the most impactful application is expected to be with PET/MR imaging, where a pseudo-CT could be generated directly for PET NAC data, requiring no additional time to acquire an attenuation correction scan, improving PET/MR workflow.

This study has several limitations. First, the training group was selected from a clinical database. While the included patients accurately reflect a population of subjects undergoing PET, the group was not selected to be optimal for healthy volunteers with no known pathology. However, the data is representative of a realistic patient population. With the addition of an increased number of datasets in the training data, it is expected that even greater resilience to this potential source of bias (which was not observed herein) could be achieved. A second limitation is that the trained model is only applicable to ^18^F-FDG PET data. Development of models for other radiotracers would require training sets of PET/CT data utilizing that specific tracer. This could be potentially limiting in tracers that are highly specific and do not have a sufficiently global distribution, as it would be expected that the deep learning model would require patterns of global physiological uptake to fully train the model. Furthermore, the model was trained on ^18^F-FDG PET data that was acquired 60 min after the administration. Utilization of deepAC with agents that have significant changes in regional uptake based and rapid pharmacokinetics may also be challenging. However, in the context of clinically routine ^18^F-FDG PET imaging as demonstrated herein, deepAC performs considerably well. Third, for the ROI approach used herein to assess PET reconstruction error, the computed error is averaged within the entire volume of the ROI. In certain regions of the brain, the error may not be distributed uniformly, and may actually be larger near the interfaces of air, bone, and soft tissue. For example, the cortical regions of the brain that are near the skull may have a larger error relative to the regions further inside the skull. However, these errors are perhaps better depicted visually, as can be seen in Figs. 5 and 6. Fourth, the current study implemented 2D CNN for pseudo-CT generation from NAC images. Potential improvement for the generation quality might be achieved using methods considering 3D image context, such as using 3D CNNs or augmented 2D CNNs with 3D spatial refinement [7, 17, 18, 31]. Finally, the pseudo-CT generated by deepAC is not a replacement for a diagnostic CT. While it may provide an anatomical reference, it does not necessarily reflect the true underlying tissue contrast. For studies that require a true anatomical reference, additional imaging must be performed. In particular, we expect extensions of deepAC for PET/MR applications, where anatomic imaging is readily available, to be particularly valuable.

## Conclusions

We have demonstrated a deep learning approach to produce accurate quantitative PET imaging by using only NAC ^18^F-FDG PET images. Such approaches will likely have a substantial impact on future work in PET, PET/CT, and PET/MR studies to reduce ionizing radiation dose and increase resilience to subject misregistration between the PET acquisition and attenuation map acquisition.

## Abbreviations

^18^F-FDG: ^18^F-fluorodeoxyglucose
BN: Batch normalization
CED: Convolutional encoder-decoder
CNN: Convolutional Neural Network
CT: Computed tomography
deepAC: Deep attenuation correction
MAE: Mean absolute error
MR: Magnetic resonance
NAC: Non-attenuation-corrected
PET: Positron-emission tomography
ReLU: Rectified linear unit
VGG16: Visual Geometry Group 16
